# Pharmacological Chaperone Therapy for Pompe Disease

**DOI:** 10.3390/molecules26237223

**Published:** 2021-11-29

**Authors:** Marc Borie-Guichot, My Lan Tran, Yves Génisson, Stéphanie Ballereau, Cécile Dehoux

**Affiliations:** SPCMIB, UMR5068 CNRS-Université Paul Sabatier-Toulouse III, 118 Route de Narbonne, F-31062 Toulouse, France; marc.borie-guichot@univ-tlse3.fr (M.B.-G.); tran@chimie.ups-tlse.fr (M.L.T.); genisson@chimie.ups-tlse.fr (Y.G.); ballereau@chimie.ups-tlse.fr (S.B.)

**Keywords:** Pompe disease, lysosomal storage disease, pharmacological chaperone

## Abstract

Pompe disease (PD), a lysosomal storage disease, is caused by mutations of the GAA gene, inducing deficiency in the acid alpha-glucosidase (GAA). This enzymatic impairment causes glycogen burden in lysosomes and triggers cell malfunctions, especially in cardiac, smooth and skeletal muscle cells and motor neurons. To date, the only approved treatment available for PD is enzyme replacement therapy (ERT) consisting of intravenous administration of *rh*GAA. The limitations of ERT have motivated the investigation of new therapies. Pharmacological chaperone (PC) therapy aims at restoring enzymatic activity through protein stabilization by ligand binding. PCs are divided into two classes: active site-specific chaperones (ASSCs) and the non-inhibitory PCs. In this review, we summarize the different pharmacological chaperones reported against PD by specifying their PC class and activity. An emphasis is placed on the recent use of these chaperones in combination with ERT.

## 1. Introduction

Pompe disease (PD), also called type II glycogen storage disease (GSDII, OMIM #232300), is a rare autosomal recessive disorder discovered in the 1960s by the Dutch pathologist Joannes Cassianus Pompe. This disorder belongs to the family of lysosomal storage diseases (LSDs) [1]. PD is caused by mutations of the *GAA* gene inducing a deficiency in the acid alpha-glucosidase (GAA) (EC 3.2.1.20), which catalyzes the hydrolysis of glycogen to glucose units in lysosomes. The prevalence of the disorder is estimated to be 1–9 out of 100,000 live births depending on ethnicity and the geographic region [2]. More than 500 mutations are known, including insertions, deletions, splice site, nonsense and missense mutations [3,4]. The severity of the disease is linked to the nature of the mutations, the level of the residual enzyme activity and the age at the onset. The accumulation of glycogen within lysosomes triggers cell malfunctions especially in cardiac, smooth and skeletal muscle cells and motor neurons. Two broad clinical phenotypes are thus distinguished [5]. The severe classic infantile onset (IOPD) type begins before the age of one year, and the associated residual enzyme activity is less than 1%. The milder late onset (LOPD) type with a higher enzyme activity appears in childhood, adolescence or adulthood. Phenotype in IOPD is a rapidly progressive cardiomyopathy, hypotonia and respiratory distress. If left untreated, death occurs in childhood. Neurological impairments in a number of patients with IOPD due to the accumulation of excess glycogen in brain have also been observed [6].

The GAA protein belongs to the glycoside hydrolase family 31 (GH31), which includes alpha-galactosidase, glucoamylase, sucrase-isomaltase and isomaltase [7]. GAA is an exo-hydrolase and possesses two aspartic acid residues acting as a nucleophile catalytic and general acid/base [8]. The *GAA* gene (GenBank NM_000152) encodes the 896-aminoacids protein weighing 110 kDa including seven glycosylation sites. GAA is glycosylated in the endoplasmic reticulum (ER) and phosphorylated on its mannose residues in the Golgi to allow the targeting of the mannose-6-phosphate receptors (M6PR). GAA undergoes a series of proteolytic and *N*-glycan processing events in the late endosomal/lysosomal compartment that yield a mature active form composed of four tightly associated peptides [9]. The mature lysosomal protein weighs 76–70 kDa and possesses a higher affinity for the glycogen than the premature forms. Recently, the structure of a recombinant human GAA was determined by X-ray crystallography [8].

A type of M6PR called cation independent-M6PR is also present on the cell surface. Its function is to capture any mannose 6-phosphate tagged enzymes that have accidentally entered the secretory pathway. Once it binds to a lysosomal enzyme, the receptor becomes internalized rapidly, and the tagged enzyme can reach the lysosome. The discovery of this naturally occurring correction opened the way to the enzyme replacement therapy (ERT) which consists of administrating a recombinant protein to compensate for the endogenous deficient enzyme [10]. In 2006, recombinant human *rh*GAA obtained marketing approval in Europe and the United States (alglucosidase alfa, marketed by Genzyme as Lumizyme™ in the USA and as Myozyme™ elsewhere). ERT is currently the only approved treatment against PD. Many clinical trials have been performed to evaluate the efficiency of ERT against PD. Although the results varied widely depending on the study and the patients, there is no doubt that ERT slows disease progression and increases life expectancy [11,12]. The most notable effect of ERT is observed on cardiac function, regardless of the disease severity. In contrast, the skeletal muscle response is variable and less important despite the high dosage in recombinant protein compared to treatments of other LSDs [13,14,15,16]. These observed limitations are at least partially due to insufficient uptake into disease-relevant tissues [17]. This therapy is also ineffective in treating neurological disorders because of enzymes’ inability to cross the blood–brain barrier. Moreover, severe anaphylactic and immunologic reactions are sometimes also observed upon ERT treatment [18,19]. The instability of the *rh*GAA in blood pH is another factor that explains the poor delivery of ERT to the muscle. To improve protein delivery, the design of the next ERT generation is under progress. NeoGAA is a new *rh*GAA developed by Genzyme that is chemically modified to include bis-phosphorylated oligosaccharides. Improvement in walking and breathing was obtained with NeoGAA, and these phase 3 clinical trial data allowed its recent approval for LOPD (avalglucosidase alfa-ngpt, marketed as Nexviazyme™ in the USA) [20].

Alternative therapeutic strategies against PD, independent or complementary to ERT, are thus still needed. Among the therapies currently explored, gene therapy and substrate reduction therapy should be mentioned [21,22,23]. This review will focus on another promising strategy, pharmacological chaperone (PC) therapy. This strategy aims at restoring the activity of the deficient enzyme with ligands stabilizing the protein by binding. After defining PC therapy, we intend to review the different pharmacological chaperones reported against PD. To achieve this, we implemented the following bibliographic methodology to survey the literature. Three bibliographic databases were used: Scifinder (https://scifinder.cas.org, accessed on 2 September 2021), Web of Science (https://apps.webofknowledge.com, accessed on 2 September 2021) and Pubmed (https://www.ncbi.nlm.nih.gov/pubmed/, accessed on 2 September 2021). The chosen keywords were: “alpha glucosidase”, “lysosomal” and “chaperone”. From the obtained references, we only kept those referring to human lysosomal alpha-glucosidase and mentioning a pharmacological chaperone.

## 2. What Is PC Therapy?

The first example of PCs was reported in 1999 by Fan and coll. [24]. In this seminal article, the authors reported that 1-deoxy-galactonojirimycin (DGJ), a potent competitive inhibitor of alpha-galactosidase A (α-Gal A), the deficient protein in Fabry disease (FD), effectively enhanced protein activity in Fabry lymphoblasts when administrated at subinhibitory concentrations. DGJ (Galafold™) has been marketed since 2016 against FD and is, to date, the only marketed PC [25].

Since this pioneering work, the concept of PC, also called pharmacoperones or pharmacochaperones, was developed and extended to a large range of conformational diseases including LSDs [26,27,28,29,30]. The word ‘chaperone’, broadly defined, applies to a molecule able to assist a protein in recovering its correct conformation. However, PCs differ from other chaperones (molecular or chemical) in their ability to specifically bind to a protein. PCs prevent retention and/or degradation by the ER quality-control system of a misfolded protein and facilitate its trafficking into organelles. This capacity to induce or stabilize the correct conformation of the misfolded protein allows increasing cellular enzyme activity. An important prerequisite for this strategy is, however, the expression of a mutated enzyme that retains some degree of catalytic activity. Compared to ERT, PC therapy offers the advantages of low molecular weight active ingredients, such as a better bioavailability, a possible blood–brain barrier crossing and oral administration. Moreover, the restoration of only 10–20% of protein activity would be, in most cases, enough to prevent clinical manifestations of the disease [31].

The first class of PCs, including Galafold™, is composed of active site-specific chaperones (ASSCs), which are competitive inhibitors of the protein used at concentrations below those usually required for cellular inhibition [32]. Numerous ASSCs have been described for a wide range of conformational diseases [27]. However, the PC has to be displaced by the endogenous protein ligand to restore protein activity, and the main limitation of the ASSCs lies in the difficulty to control the balance between protein inhibition and activation. To circumvent this issue, a second class of PCs has emerged that is composed of non-inhibitory PCs. These compounds behave as PCs by increasing residual enzyme activity, but they have the advantage of presenting no (or a weak) inhibition. As for ASSCs, the stabilization mechanism of these allosteric compounds requires a sufficient binding affinity to increase the stability of the mutant enzyme. Even though many examples of non-inhibitory PCs have been reported for various conformational diseases, there are no commercially available compounds at this time [33].

The efficacy of the PC therapy is highly dependent on the enzyme variants. By definition, PCs are not useful when the protein is absent because the gene is affected by a deletion, a stop gain mutation, a splicing mutation or a mutation occurring in the regulatory region. The use of PCs is thus limited to the case of missense mutations. Moreover, while some enzyme variants appear to respond quite well, others show only minor net increases in activity, lysosomal trafficking and enzyme processing/maturation (reference [34] and references therein).

Recent studies showed that chaperones may also be able to increase the stability of the endogenous wild-type enzymes, although PC therapy was initially designed to rescue mutant proteins [35]. Moreover, preclinical studies showed that PCs also improve enzyme stability, lysosomal trafficking and/or activity in cultured Pompe [36,37,38], Fabry [39] and Gaucher cells [40] incubated with the exogeneous recombinant enzymes. This observation has given rise to a new application of PCs known as combination therapy. This consists of not in increasing the residual activity of a mutant enzyme, but in increasing the efficiency of the recombinant enzymes used for ERT by combining ERT and PC therapy. Thus, the use of PCs is no longer limited to some enzyme variants but is extended to the recombinant enzymes administered to patients treated with ERT.

## 3. Overview of Pharmacological Chaperones of GAA

Since the emerging of PC therapy, numerous chaperones have been reported, mainly for Gaucher disease (GD) and FD. This is probably because of its lower prevalence compared to FD and GD, so fewer studies have dealt with PD. Yet, 297 different missense GAA mutations are known to cause PD [3]. These mutations would concern about 10–15% of Pompe patients that would thus be amenable to PC therapy [31]. The discovery of new PCs of GAA is therefore an important therapeutic issue in the fight against PD.

### 3.1. Sugar- and Iminosugar-Based Chaperones

To identify PCs, research works initially relied on known alpha-glucosidase inhibitors. In the search for new antidiabetic agents, numerous iminosugars have showed inhibitory effect against alpha-glucosidases [41]. Thus, the first PCs of GAA are found mostly in iminosugars family. Some also belong to the carbohydrate family. As the protonated forms of iminosugars mimic the transition state of the glucopyranoside hydrolysis, they behave mostly as competitive inhibitors of glycosidases [42]. The major inconvenience of iminosugars as glycosidase inhibitor is their lack of selectivity.

#### 3.1.1. DNJ and DNJ Derivatives

**Figure 1:**
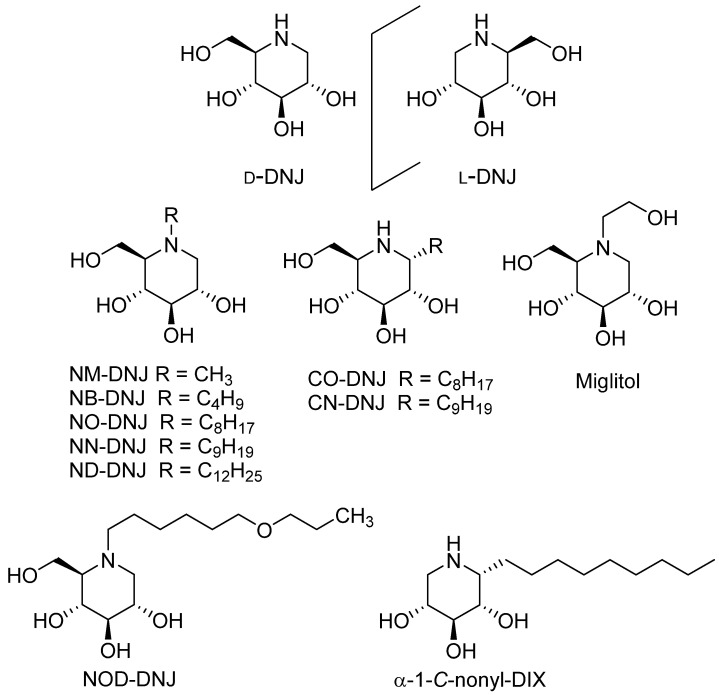
Structures of *N*-alkyl and *C*-alkyl derivatives of DNJ: d-DNJ and l-DNJ, *N*-methyl-DNJ (NM-DNJ), *N*-butyl-DNJ (NB-DNJ), *N*-octyl-DNJ (NO-DNJ), *N*-nonyl-DNJ (NN-DNJ), *N*-dodecyl-DNJ (ND-DNJ), *C*-octyl-DNJ (CO-DNJ) and *C*-nonyl-DNJ (CN-DNJ), *N*-(7-oxadecyl)-DNJ (NOD-DNJ), *N*-hydroxyethyl-DNJ (miglitol) and α-1-*C*-alkyl-d-xylitol (α-1-*C*-nonyl-DIX).

Deoxynojirimycin (DNJ) and its derivatives are the most studied compounds as potential chaperones of GAA (Figure 1). DNJ and congeners, in which the configuration of all the hydroxyl groups mimics those of d-glucopyranose, inhibit alpha-glucosidases and beta-glucosidases in a competitive and reversible manner [43]. A famous example of an alpha-glucosidase inhibitor is the approved anti-diabetic drug, *N*-hydroxyethyl-DNJ (miglitol, marketed as Glyset™), which allows the control of sugar level in blood [44]. The inhibition of lysosomal GAA with DNJ and its derivatives was also extensively explored, and not surprisingly, numerous derivatives have been shown to be GAA inhibitors. A non-exhaustive list includes: DNJ, its (l) enantiomer, various *N*-alkylated derivatives including *N*-butyl-DNJ (NB-DNJ) and *N*-nonyl-DNJ (NN-DNJ), miglitol and *N*-[β-(4-ethoxycarbonylphenoxy)-ethyl]-DNJ (emiglitate) (Figure 1) [37,45,46,47].

The first observation of a chaperone activity for GAA was performed by Asano and coll. in 2006 [46]. In the course of the search for new chaperones against GD [48], the authors reported the inhibitory and chaperoning activities of *N*-alkyl and *C*-alkyl DNJ observed with beta-glucocerebrosidase (β-GCase) compared to GAA. The four most potent inhibitors of β-GCase, NO-DNJ, NN-DNJ, CO-DNJ and CN-DNJ, inhibit moderate GAA (IC_50_ 1.5–5 µM). The ability of these four compounds to enhance activity of β-GCase and GAA in Gaucher fibroblasts was investigated. In N370S Gaucher fibroblasts, an increase in β-GCase was induced by the four compounds, whereas a weak GAA activation was observed upon the administration of CO-DNJ and CN-DNJ (Table 1). Of note, the same authors also grafted several *N*-alkyl DNJ on a β-cyclodextrin core [49]. The 7-valent and 14-valent NN-DNJ showed the strongest inhibition for β GCase (respectively, IC_50_ 8 nM and 96 nM) as well as for GAA (respectively, IC_50_ 10 nM and 13 nM). However, no enhancement of GAA activity was observed after the incubation of the 7-valent compound on N370S and L444P GD fibroblasts.

The authors also developed an α-1-*C*-alkyl-d-xylitol (α-1-*C*-alkyl-DIX, Figure 1) series built on a simplified DNJ scaffold [55]. To assess the selectivity of these *C*-alkyl derivatives, compounds were evaluated for inhibition and chaperoning abilities vs. β-GCase as well as GAA. Whereas α-1-*C*-nonyl-DIX is a nanomolar inhibitor of β-GCase (Ki 2.2 nM), no GAA inhibition was found up to 1 mM. Accordingly, the α-1-*C*-nonyl DIX induced only a weak enhancement of GAA activity in the Gaucher patient fibroblasts (1.2-fold increase, 5–50 µM) (Table 2).

After this pioneering work proving the possibility of a GAA activation by DNJ derivatives, several studies were performed to investigate the GAA chaperoning mechanism. In 2007, Andria et al. studied the action of DNJ and NB-DNJ on fibroblasts of eight Pompe patients with different genetic backgrounds and different phenotypic presentations [50]. The administration of both compounds at 20 µM on fibroblasts carrying the L552P and G549R mutations led to a significant increase in GAA activity (1.3–7.5-fold) (Table 1) [50]. These results were confirmed with assays on HEK293T cells presenting the same mutations. Moreover, Western blot analyses showed the presence of substantial amounts of the mature and active 76–70 kDa GAA forms after iminosugar administration. The immunofluorescence assays in cells overexpressing the L552P mutation showed an enhancement of trafficking and delivery of mutated GAA to lysosomes by DNJ and NB-DNJ. Authors concluded that the stimulation of endogenous residual GAA activity is an alternative therapeutic approach for the PD patients.

Do et al. extended these studies by characterizing the effect of DNJ on 14 Pompe patient-derived fibroblasts with at least one missense mutant allele on 76 different mutant forms of GAA generated by site-directed mutagenesis and transiently expressed in COS-7 cells [61]. Among the 14 cell lines, 6 showed a significant enhancement of GAA activity ranging from 4- to 18-fold. Moreover, on the 76 mutant GAA, 16 were responsive to DNJ, showing improvement in trafficking to the lysosome. To rationalize the link between mutation location and responsiveness to DNJ, a structural model of human GAA was constructed. The authors noticed that for the 16 DNJ-responsive variants (except P285 and M519), the mutations were located more than 10 Å away from the active site. To complete the investigations on DNJ, Khanna et al. performed an in vivo study with a new transgenic mouse model expressing the human P545L mutant on a *Gaa* KO background. Their results demonstrate that DNJ treatment resulted in significant glycogen reduction in disease-relevant tissues [62].

In 2007, Okumiya et al. investigated the action of DNJ and four *N*-alkyl derivatives thereof (NOD-DNJ, ND-DNJ, NN-DNJ and NB-DNJ) as well as voglibose and acarbose (Table 1) in PD patients’ fibroblasts [51]. Four mutant phenotypes were chosen, in which the GAA synthesis was normal, but the post-translational modifications were incorrect: Y455F/Y455F, P545L/P545L, 525del/R600C and D645E/R854X. Two cell-lines (Y455F/Y455F and P545L/P545L) showed a significant increase in GAA activity in the presence of DNJ at 100 µM. Among the DNJ derivatives, NB-DNJ (and NOD-DNJ to a lesser extent) was the most effective in restoring the GAA activity, whereas the long alkyl chain derivatives NN-DNJ and ND-DNJ caused a loss in activity. Western blot analysis confirmed that NB-DNJ increases the amount of mature GAA molecular forms, which is in accordance with results of Andria et al. [50]. Immunocytochemistry further showed that NB-DNJ improved the transport from the ER to the lysosome of mutant enzymes.

An important step forward was achieved with the results of Parenti et al., published in 2009 [36]. Indeed, the authors demonstrated that the co-incubation of *rh*GAA and NB-DNJ increases enzyme activity correction with a synergic effect (Table 3). Their investigations were based on preliminary results showing that the preincubation of recombinant β-GCase and IFG improved uptake and stability in Gaucher cells [40]. First, Parenti et al. demonstrated that the co-administration of NB-DNJ and *rh*GAA led to an improvement of the GAA activity correction in fibroblasts from PD patients and also in vivo in a KO mouse model of the disease. NB-DNJ treatment was effective in improving the delivery of *rh*GAA to the lysosomal compartment, as indicated by colocalization studies, to increase the amounts of mature GAA polypeptides by Western blot analysis and to stabilize *rh*GAA in PD fibroblasts. This therapeutic advance was particularly important for patients poorly responding to ERT due to an insufficient level of *rh*GAA in the tissues. Besides, until now, the use of PCs had only been possible for the treatment of patients with missense mutations, causing an alteration in the conformation of the enzymatic proteins that retain their catalytic activity, i.e., only about 10–15% of patients [36]. Importantly, with this study, the use of PC became relevant to all patients undergoing ERT with *rh*GAA. The authors extended their work to FD and showed that this enzyme activity enhancement was also observed with combination of DNJ and recombinant alpha-galactosidase in FD fibroblasts. Amicus Therapeutics performed a clinical trial evaluating the efficacy and safety of AT-GAA, a next generation enzyme (cipaglucosidase alfa identified as ATB200) in combination with a chaperone molecule (NB-DNJ identified as AT2221) in the late-onset form of PD until 2023 [63,64]. The U.S. Food and Drug Administration (FDA) very recently accepted to review Biologics License Application (BLA) and the New Drug Application (NDA) for AT-GAA combination [65].

Other studies have shown that the same synergy between ERT and PC therapy could be reached with DNJ. After having shown that DNJ stabilizes *rh*GAA and prevents the loss of activity in vitro and in human blood ex vivo, Khanna et al. demonstrated that the coadministration of DNJ and *rh*GAA increases the stability of the recombinant protein in vivo in rats and in KO mice [38]. They went even further, showing that the co-administration increases the GAA activity in disease-relevant tissues such as the heart and skeletal muscles of GAA KO mice compared to administration of *rh*GAA alone. This preclinical proof of concept led to a clinical trial performed with 25 Pompe patients [66]. This phase 2a study consisted of an open-label, fixed-treatment sequence with different doses of DNJ (from 50 mg to 600 mg) to evaluate their effects on the pharmacokinetics and tissue levels of intravenously administrated *rh*GAA (Alglucosidase alpha, Genzyme). The co-administration led to a 1.2- to 2.8-fold increase in total GAA activity and protein in plasma compared to *rh*GAA alone administrated in all 25 Pompe patients. Muscle GAA activity was also increased for all co-administration treatments.

During their research program aiming at exploring the stereoselectivity in biomolecular recognition processes, D’Alonzo et al. investigated the chaperoning potential of the l-enantiomer of DNJ (Figure 1) [37]. Indeed, several l-iminosugars proved to be weaker and non-competitive glycosidase inhibitors [67,68,69,70,71,72] than their enantiomers, and some of them (either alone or in combination with the d-enantiomers) have a significant chaperoning activity on various misfolded glycosidases [71,73,74,75]. Authors performed the first stereocontrolled synthesis of l-DNJ and l-NB-DNJ. Inhibition assays on various glycosidases showed that l-NB-DNJ was a more modest inhibitor than its enantiomer. Interestingly, no *rh*GAA inhibition was found at 1 mM. Moreover, l-NB-DNJ led to an increase in GAA activity in fibroblasts of PD patients carrying the mutation p.L552P/p.L552P (Table 2). Notably, the activating effect was higher than that observed for d-NB-DNJ (1.5-fold increase instead of 1.3 at 20 µM). However, this activity enhancement is insufficient to stimulate therapeutic application. In addition, the coincubation of l-NB-DNJ with *rh*GAA led to a comparable synergistic enhancing effect to that observed with d-DNJ. l-NB-DNJ, and thus it represents a promising candidate for combination therapy (Table 3).

#### 3.1.2. Other Iminosugars: Pyrrolidine and Pyrrolizidine

**Figure 2:**
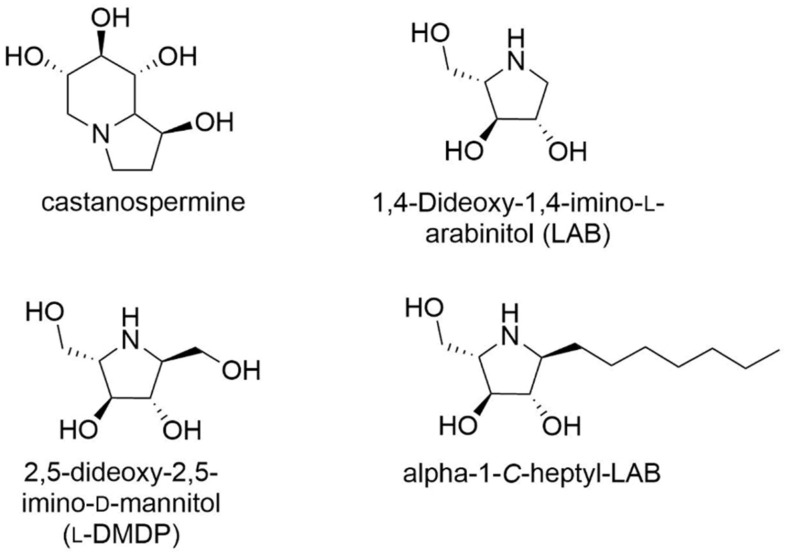
PC with a pyrrolizidinic or pyrrolidinic structures.

DNJ derivatives are mid to potent GAA inhibitors, but they suffer from a lack of selectivity towards GAA by inhibiting alpha-glucosidases from other sources. To identify new GAA chaperones, in 2012, Heikinheimo et al. compared the GAA inhibition, stabilization and activity enhancement of 13 glucosidase inhibitors including DNJ derivatives, castanospermine (Figure 2) and few sugars (see Section 3.1.3) [76]. First, inhibition assays were established on GAA, and the three most potent inhibitors with a sub-micromolar K_i_ were d-DNJ (K_i_ 0.25 µM), NN-DNJ (K_i_ 0.49 µM) and castanopermine (K_i_ 0.68 µM). The selected inhibitors were studied on the cellular localization on PD mutations (p.A445P, p.G549R and p.L552P) and wild-type GAA (*wt*GAA). DSF studies showed that the three best stabilizers at a micromolar concentration, not only at acidic pH (pH 4.3) but also neutral pH, were NN-DNJ, castanopermine and NM-DNJ (*N*-methyl-DNJ). The best inhibitors, including castanospermine, also improved the lysosomal targeting of the disease-associated GAA variant. In the absence of a protein crystal structure at that time, a homology model of GAA based on the X-ray structure of human maltase-glucoamylase (MGA; PDB ID: 2QMJ) was built to rationalize these results. Ligand docking not only pointed out the significance of having three properly oriented hydroxyl groups accounting for specific binding, but also that the GAA active site is a wide pocket allowing to bind large ligands, such as castanospermine. However, to date, the evaluation of castanospermine as chaperone has not been continued.

In 2020, Kato et al. reported a strategy for designing selective GAA inhibitors vs. ER α-glucosidase II [77]. The superimposition of the structures of GAA (PDB ID: 5NNJ) and ER alpha-glucosidase II (PDB ID: 5IEE), co-crystallized with DNJ, showed a high similarity to the glucose-binding site. 1,4-Dideoxy-1,4-imino-l-arabinitol (LAB, Figure 2) is a well-known α-glucosidase inhibitor [78,79,80,81]. α-1-*C*-Heptyl-LAB was the most potent GAA inhibitor and also the most selective one (IC_50_ 0.44 µM, selectivity index of 168.2). This result was rationalized by molecular dynamic simulation experiments using the crystal structure of GAA with DNJ (PDB ID: 5NN5), highlighting the key role of the alkyl chain length in the stabilization of the ligand-binding conformation. Docking studies showed that the size of the chain storage pocket is narrower in ER alpha-glucosidase II than in GAA. A temperature denaturation assay showed that α-1-*C*-heptyl-LAB is more efficient in stabilizing GAA. However, the increased activity of these compounds has not been evaluated in Pompe patient cells. The current results are therefore insufficient for the moment to identify α-1-*C*-Heptyl-LAB as a PC of GAA.

#### 3.1.3. Carbohydrates

**Figure 3:**
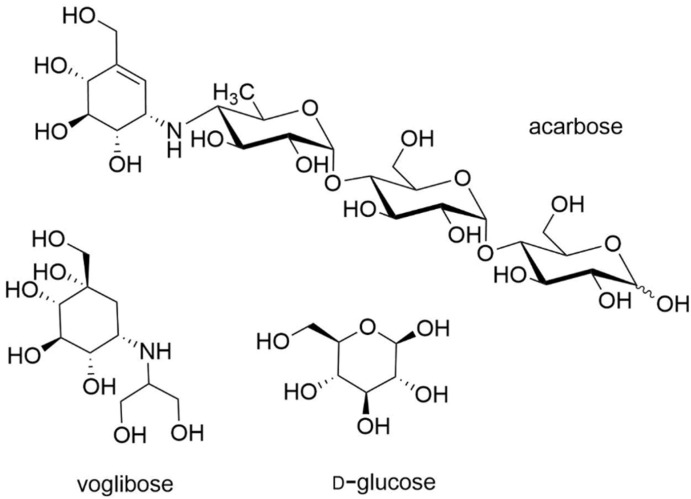
Carbohydrates evaluated as GAA PC.

In 2006, Kakavanos et al. were interested in increasing *rh*GAA production in CHO-K1 cells to facilitate the access to *rh*GAA for ERT. First, they showed that d-Glucose (Figure 3) is a weak competitive inhibitor of GAA (K_i_ 44 mM) [52]. In the presence of 6.0 g.L^−1^ glucose or galactose, the authors noticed an increase in GAA production in a CHO-K1 cell and a preservation of the enzymatic activity for more than 4 days (Table 1). Moreover, the same concentration of glucose allowed an increase in GAA activity in the fibroblasts of adult-onset PD patients. In 2007, Reuser et al. found that acarbose increased slightly GAA activity (Y455F/Y455F, 1.4-fold; and P545L/P545L, 1.9-fold), unlike voglibose, which did not show chaperone behavior (Table 1) [51]. Heikinheimo et al. evaluated the ability of few sugars including d-glucose, acarbose and voglibose (Figure 3) to restore localization of three GAA variants, including both mild (p.G549R) and severe (p.L445P, p.L552P) phenotypes [76]. Even large concentrations of d-glucose and acarbose failed to restore the lysosomal localization of the p.L552P variant, although they could restore the milder p.G549R variant in lysosome. However, voglibose was effective in restoring the lysosomal localization of all tested GAA variants, but no evaluation of the enzymatic activity increase was carried out.

### 3.2. Ambroxol and Analogs

**Figure 4:**
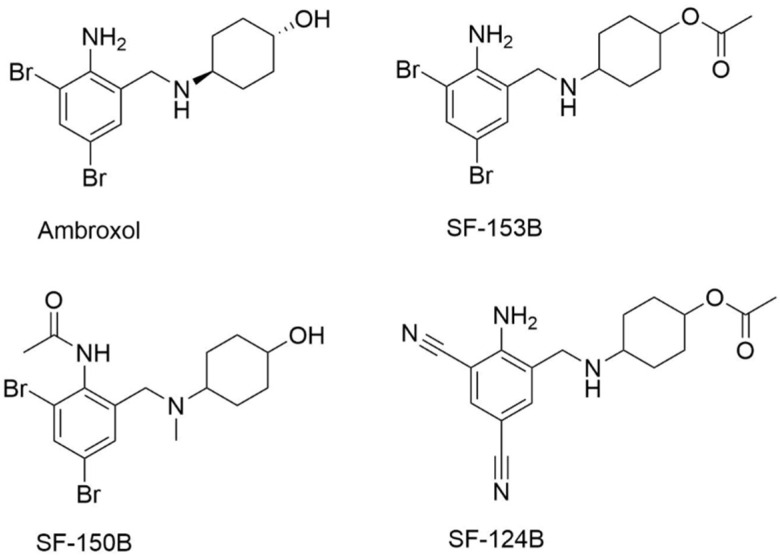
Ambroxol and its derivatives.

Starting from the observation that PCs, like NB-DNJ, display little selectivity for the glycosidase enzyme family and often bind to various different lysosomal hydrolases, Lukas et al. evaluated the capacity of several known PCs, especially for GD, to enhance the activity of GAA [82]. They investigated the effect of the expectorant Ambroxol (Figure 4), identified as a PC for GD [83], on mutant GAA.

The mutant forms, expressed in HEK-293H cells, were selected for their ability to respond to PC treatment: NB-DNJ for p.Y455F, p.P545L [51] and p.L552P [50] and DNJ for p.Y575S [61]. While Ambroxol alone has no effect on the tested mutant forms, the combination of Ambroxol and NB-DNJ induces an increase in activity for the p.L552P mutation (2.2-fold increase compared to DNJ alone treatment) (Table 4). Similarly, when used in combination with DNJ, Ambroxol causes a significant improvement in the enzyme activity for mutants p.Y455F (1.6-fold), p.P545L (2.3-fold) and p.L552P (2.2-fold). It remains unclear why the DNJ-Ambroxol combination displays a synergistic effect on mutants p.Y455F and p.P545L while NB-DNJ-Ambroxol combination does not. No synergistic effect was observed for mutation p.Y575S. The authors admit that the utilized cell culture system cannot allow for deciphering the likely complex mode of action of Ambroxol.

Several structural analogs of Ambroxol were also tested by Lukas et al. according the same protocol on p.Y455F and p.L552P mutants [84]. Among these, the *O*-acetylated form of Ambroxol (SF-153B, Figure 4) in combination with DNJ induces a significant increase in activity compared to DNJ treatment alone for p.Y455F (2-fold increase, value estimated from reference [84]) and p.L552P (5.5-fold increase) mutants. A marked synergistic effect was also observed on p.L552P mutant with the *N*-acetylated-*N*-methylated analog of Ambroxol SF-150B (Figure 4) and with the dicyano analog SF-124B (Figure 4) (2.8-fold compared to DNJ alone for both compounds).

### 3.3. Aminoacids

**Figure 5:**
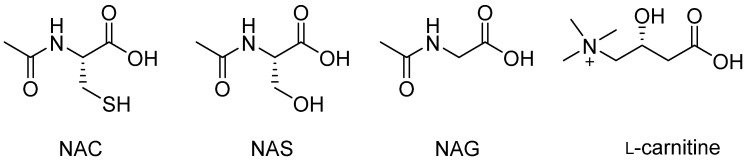
Aminoacids acting as non-inhibitory PCs.

In 2012, Parenti et al. reported that *N*-acetyl-l-cysteine (NAC), *N*-acetylglycine (NAG) and *N*-acetyl-l-serine (NAS) are non-inhibitory allosteric pharmacological chaperones of GAA (Figure 5) [56,57]. First, the authors showed in vitro that NAC, NAG and NAS allowed the rescue of *rh*GAA activities at a pH of 7.0. The largest effect was obtained using NAC with ca. 90% of *rh*GAA activity retention at 10 mM after 48h of incubation (compared to 75% and 65% for NAS and NAG, respectively). An enhancement of endogenous GAA residual activity was also observed in presence of NAC in COS7 cells expressing the three mutant proteins L552P, A445P and Y455F (Table 2). Moreover, the co-incubation of *rh*GAA with NAC in PD fibroblasts led to an increase in the residual activity of mutated GAA ranging from 3.7–8.7-fold compared to the administration of *rh*GAA alone for all tested genotypes (Table 3). Subsequently, in vivo assays in a mouse model showed an increase in GAA activity in all tissues from subjects treated with ERT and NAC compared to subjects treated with ERT alone [56,57]. They also showed that NAC and its derivatives are not *rh*GAA inhibitors. This analysis was first confirmed with in silico modeling and molecular dynamics studies of GAA and later on with the co-crystallization of NAC in two allosteric sites of *rh*GAA (pdb code: 5NN4) [8]. However, it is important to note that allosteric PCs stabilize GAA at higher concentrations (about mM) than the concentrations typically used with ASSCs (in the micromolar range). The synergistic effect of a combination of two chaperones and ERT therapy was also evaluated. First, it was shown that the simultaneous use of NAC (10 mM) and NB-DNJ co-incubated with *rh*GAA significantly increases the stability of the enzyme (+10 °C) compared to NB-DNJ alone. Moreover, the simultaneous use of NAC and NB-DNJ as an adjunct to ERT treatment increased residual GAA activity in two fibroblast patient cell lines (Table 3).

Very recently, Iacono et al. showed that l- and d-carnitine as well as acetyl-d-carnitine displayed similar allosteric GAA activation (Figure 5) [58]. l-carnitine increases the GAA residual activity in fibroblast presenting the p.L552P mutation (Table 2). Moreover, the co-incubation of l-carnitine (tested in a range of 1 to 20 mM) with *rh*GAA in three different fibroblast cell lines also showed an increase in residual GAA mutant activity for all cell lines with a dose-dependent effect (Table 3). These results were confirmed by Western blot experiments, which showed an increase in the amount of mature GAA isoforms compared to ERT treatments alone [58]. These in vitro and in vivo studies showed that the use of acetylated amino acids, including NAC or l-carnitine, in addition to ERT treatment increases the efficiency of ERT on various GAA variants, and appears to be a promising tool for the treatment of PD.

### 3.4. Compounds Identified by HTS

As we have seen, a large part of GAA pharmacological chaperones consists of sugar or iminosugar derivatives displaying a low specificity and a relatively short half-life times in cells [85]. To address these limitations, the NIH Chemical Genomics Center initiated a quantitative high-throughput screening (qHTS) campaign in the late 2000s in order to identify non-sugar small molecule PCs for LSDs. In 2009, Motabar et al. developed an assay to test the hydrolytic capacity of GAA in spleen homogenate for HTS screening [86]. This qHTS of more than 200,000 compounds allowed for the identification of two series of GAA activators.

#### 3.4.1. ML201 and Its Derivative CID36649951

**Figure 6:**
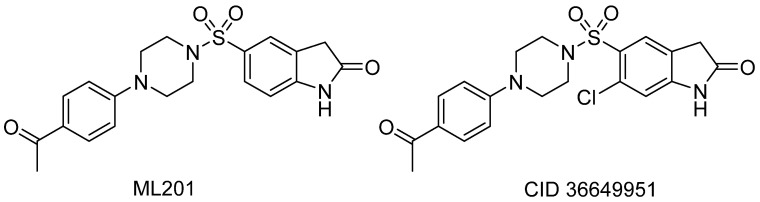
ML201 and its derivative CID36649951.

The first lead compound uncovered by means of this qHTS was 5-(4-(4-acetylphenyl)piperazin-1-ylsulfonyl)indolin-2-one, or ML201 (Figure 6), which was identified as a selective inhibitor of GAA, inactive against other glycosidase enzymes (α-galactosidase A or β-GCase) [53,54]. ML201 exhibits an IC_50_ value of 0.94 µM in a red dye-tissue homogenate assay [54,86]. Despite this significative inhibitory activity, which is undesirable for a PC, ML201 also presents the ability to thermostabilize GAA.

An extensive SAR study highlighted another compound of this family, the chlorinated analog CID 36649951 (Figure 6), which displays an IC_50_ value against GAA (1.63 µM) in the same range as ML201 and is able to stabilize GAA against thermal denaturation as ML201 does.

These results prompted the authors to evaluate its impact on GAA translocation in lysosomes. These experiments brought to light the toxicity of ML201 for both wild-type and Pompe patient-derived fibroblasts, even at a concentration of 10 µM after a 6-day treatment. In contrast, they showed that CID 36649951 induced a translocation of GAA to the lysosomes in WT fibroblasts and in three Pompe patient cell lines at a concentration of 5 µM after a 5-day treatment. Both compounds were also able to increase the GAA activity upon 5 days of treatment for the WT fibroblasts and Pompe F2845 cell lines.

Despite these interesting results, the strong inhibitory properties of compound ML201 and its analogs reduces their potentiality as PCs. This is the reason that prompted the NIH group to pursue non-inhibitory small molecule chaperones for GAA.

#### 3.4.2. CID1512045 and ML247

**Figure 7:**
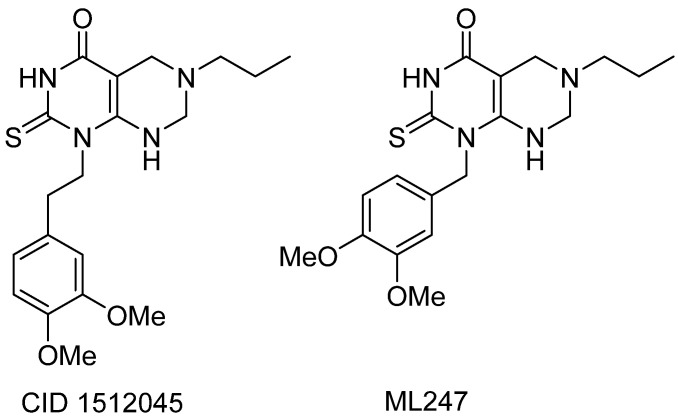
Structures of CID1512045 and ML247.

The qHTS implemented by the NIH also allowed for the identification of a new non-iminosugar activator of GAA, the lead compound CID 1512045 (Figure 7). A large SAR study showed that a small increase in efficacy could be achieved by shortening the ethyl linker by one carbon atom as in compound ML247 (Figure 7) [59,60]. The efficacy (percentage of activation at 77 µM compared to base line) and AC_50_ (concentration necessary to obtain 50% of maximal activation) were both considered when comparing the two analogs. ML247 exhibits an AC_50_ value of 6.31 µM in a red dye tissue homogenate assay [86] (16.79 µM for CID 1512045) and an efficacy of 365% (356% for CID 1512045) [59]. ML247 is not an inhibitor of GAA and did not activate other tested glycosidase enzymes (α-galactosidase A or β-GCase).

Marugan et al. also showed that ML247 increases the translocation of GAA to lysosomes in PD patient-derived fibroblasts (with around 10% translocation of p.Y455C/p.G638W mutant GAA to the lysosomes at 20 µM vs. 3% for DNJ), proving its capacity to chaperone GAA. The in vivo evaluation of ML247 showed no acute toxicity at 50 mg/Kg IP dosing and a 2.75 h half-life in plasma. ML247 was claimed to be the first non-inhibitory small molecule chaperone of GAA.

## 4. Summary and Outlook

In recent decades, important advances have been made for the understanding of the mechanism of Pompe disease [87]. Since 2006, the only, and so far, unique, approved treatment available for PD is enzyme replacement therapy (ERT) with alglucosidase alfa or avalglucosidase alfa, both marketed by Genzyme. The limitations of ERT have stimulated the scientific community to investigate alternative low molecular weight compound-based approaches against this inherited metabolic disorder [2]. PC therapy is one of the strategies actively considered to enhance the activity of misfolded variants of GAA, the deficient enzyme involved in PD.

The similarity between GAA and GBA, the glucosidase involved in GD, initially fed the search for PC molecule against PD. The first small molecules described as PC for GAA were indeed DNJ and its derivatives, firstly identified as PC for GBA. Despite their inhibitory potency dictating a narrow therapeutic window, one of those iminosugars, NB-DNJ, was recently submitted to FDA for approval for its coadministration with the avalglucosidase alfa recombinant enzyme. l-DNJ was also identified as a non-inhibitor PC in this family.

Ambroxol, another PC described for GD, is also a promising PC for GAA, especially in combination with DNJ or NB-DNJ.

A new encouraging series is constituted by some acetylated amino acids identified recently as non-inhibitory PC for GAA. In particular, *N*-acetyl-cysteine has been proven to enhance GAA residual activity and presents a synergistic effect when associated with ERT.

A large HTS implemented by the NIH in the early 2010s allowed for the identification of the non-iminosugar chaperones of GAA. Among them, CID1512045 and its optimized analogue ML247 have been proven to be promising non-inhibitory chaperones against PD. Despite these encouraging results, these compounds have not been investigating further in the literature.

In order to pursue the development of the PC therapy against PD, the discovery of new PC small molecules devoid of any inhibition towards GAA is still a challenge. However, the results obtained in the last decade, with new non-iminosugar PCs for instance, indicate that this challenge can be met. Significant advances can also be expected in the fight against PD thanks to the combination of ERT and PC. For this reason, a patent for the treatment of PD by a combination of *m*RNA encoding for the lysosomal deficient GAA and PC was recently filed [88]. This combination of PC with other therapies is undoubtedly a promising approach [2].

## Figures and Tables

**Table 1:** ASSCs for GAA.

Compound	IC_50_ µM (Ki µM)	Fold Increase	Fibroblasts	Ref.
DNJ	1.0 ^a^	1.3–7.5 at 20 µM	L552P PD	[50]
		2.5 at 20 µM	G549R PD	[50]
		5 at 100 µM ^b^	Y455F PD	[51]
		7 at 100 µM ^b^	P545L PD	[51]
NB-DNJ	5.0 ^a^	1.3 at 20 µM	L552P PD	[37]
		1.8–5.6 at 20 µM	L552P PD	[50]
		3 at 20 µM ^b^	G549R PD	[50]
		14.0 at 10 µM ^c^	Y455F PD	[51]
NOD-DNJ	1.0	7.9 at 10 µM ^c^	P545L PD	[51]
CN-DNJ	4.8 ^a^	1.3 at 5 µM	N370S GD	[46]
		1.2 at 20 µM	L444P GD	[46]
CO-DNJ	5.0 ^a^	1.2 at 5 µM	N370S GD	[46]
		1.3 at 20 µM	N370S GD	[46]
d-glucose	ND ^c^ (Ki 44 mM)	1.5 at 33 mM (6 g.L^−1^)	Adult-onset PD	[52]
Acarbose	59 and 81 ^d^ µmolL^−1^	1.4 at 50 µM	Y455F PD	[51]
		1.9 at 50 µM	P549L PD	[51]
CID 36649951	1.63	ND ^c^	F2845 PD	[53,54]

^a^ from cell lysates of normal human fibroblasts (GM00498B) (from reference [46]); ^b^ estimated from the reference; ^c^ ND: not determined; ^d^ evaluated, respectively, with precursor and mature forms of wild-type GAA.

**Table 2:** Non-inhibitory PCs for GAA.

Compound	Fold Increase	Cell Lines	Ref.
*α*-1-*C*-nonyl-DIX	1.2 at 5 µM up to 50 µM	N370S GD fibroblasts	[55]
l-NB-DNJ	1.5 at 20 µM	L552P PD fibroblast	[37]
NAC	3.0 at 10 mM	L552P COS7cell	[56,57]
	3.7 at 10 mM	A445P COS7cell	[56,57]
	3.6 at 10 mM	Y455F COS7 cell	[56,57]
l-Carnitine	3.0 at 2 mM	L552P/L552P PD fibroblast	[58]
CID 1512045	3.56 at 77 µM	Spleen homogenate	[59,60]
ML247	3.65 at 77 µM	Spleen homogenate	[59,60]

**Table 3:** PC candidates for combination therapy with ERT.

PC	Ref.
NB-DNJ	[36,63,64]
d-DNJ	[38]
l-NB-DNJ	[37]
NAC	[56]
NAC + NB-DNJ	[56]
l-carnitine	[58]
l-carnitine + NB-DNJ	[58]

**Table 4:** Combination of two PCs.

PCs in Combination	Fold Increase	Cell Line	Ref.
Ambroxol + NB-DNJ	2.2 ^a^ at 40 µM and 20 µM NB-DNJ	L552P HEK-293H	[55]
Ambroxol + DNJ	1.6 ^b^ at 40 µM and 20 µM DNJ	Y455F HEK-293H	[82]
	2.3 ^b^ at 40 µM and 20 µM DNJ	P545L HEK-293H	[82]
	2.2 ^b^ at 40 µM and 20 µM DNJ	L552P HEK-293H	[82]
SF-153B + DNJ	2.0 ^b^ at 40 µM and 20 µM DNJ	Y455F HEK-293H	[84]
	5.5 ^b^ at 40 µM and 20 µM NB-DNJ	L552P HEK-293H	[84]
SF-150B + DNJ	2.8 ^b^ at 40 µM and 20 µM DNJ	L552P HEK-293H	[84]
SF-124B + DNJ	2.8 ^b^ at 40 µM and 20 µM DNJ	L552P HEK-293H	[84]

^a^ fold increase compared to NB-DNJ monotherapy; ^b^ fold increase compared to DNJ monotherapy.
